# Greece and Turkey Shaken by African tectonic retreat

**DOI:** 10.1038/s41598-021-86063-y

**Published:** 2021-03-22

**Authors:** Jiannan Meng, Ozan Sinoplu, Zhipeng Zhou, Bulent Tokay, Timothy Kusky, Erdin Bozkurt, Lu Wang

**Affiliations:** 1 State Key Laboratory of Geological Processes and Mineral Resources, Center for Global Tectonics, School of Earth Sciences, China University of Geosciences, Wuhan, 430074 China; 2 Three Gorges Research Center for Geo–hazards, China University of Geosciences, Wuhan, 430074 China; 3 Department of Geological Engineering, Middle East Technical University, Ankara, Turkey

**Keywords:** Natural hazards, Geodynamics, Tectonics

## Abstract

Earthquakes are a consequence of the motions of the planet’s tectonic plates, yet predicting when and where they may occur, and how to prepare remain some of the shortcomings of using scientific knowledge to protect human life. A devastating Mw 7.0 earthquake on October 30, 2020, offshore Samos Island, Greece was a consequence of the Aegean and Anatolian upper crust being pulled apart by north–south extensional stresses resulting from slab rollback, where the African plate is subducting northwards beneath Eurasia, while the slab is sinking by gravitational forces, causing it to retreat southwards. Since the retreating African slab is coupled with the overriding plate, it tears the upper plate apart as it retreats, breaking it into numerous small plates with frequent earthquakes along their boundaries. Historical earthquake swarms and deformation of the upper plate in the Aegean have been associated with massive volcanism and cataclysmic devastation, such as the Mw 7.7 Amorgos earthquake in July 1956 between the islands of Naxos and Santorini (Thera). Even more notable was the eruption of Santorini 3650 years ago, which contributed to the fall of the Minoan civilization. The Samos earthquake highlights the long historical lack of appreciation of links between deep tectonic processes and upper crustal deformation and geological hazards, and is a harbinger of future earthquakes and volcanic eruptions, establishing a basis for studies to institute better protection of infrastructure and upper plate cultures in the region.

## Introduction

The Mw 7.0 Samos (Néon Karlovasion) earthquake occurred as the result of normal faulting, where the upper crust of the Aegean was pulled apart, with the initial rupture at an estimated focal depth of 11–21 km near the border between Turkey and Greece, in the Aegean Sea. The earthquake caused massive damage in Greece and Turkey, and was globally the deadliest of 2020, with 118 deaths attributed to the earthquake and associated phenomena. Effects of the earthquake included high-intensity ground shaking with associated collapse of buildings, liquefaction, rock falls, landslides, and tsunami^1,2^. This high-magnitude normal-slip earthquake, MW 7.0 (USGS) (Table 1) occurred on an E-W striking normal fault, with slip of up to 4 m on a fault estimated to be ~ 20 × 20 km in area. The initial USGS NEIC solution gave a focal depth of 21 km, while the W phase moment tensor found the focal depth of 11.5 km fit the seismic data better. AFAD (Disaster and Emergency Management of Turkey) (Table 1) suggests that the focal depth was 17.26 km. The focal mechanism solutions from different agencies are listed in Table 1.

**Table 1:** Focal mechanism solutions for the major shock and aftershocks.

Event	Lon	Lat	Mag	Depth (km)	Strike	Dip	Rake	Strike	Dip	Rake	Time (UTC)
Mainshock USGS	26.79	37.918	Mw 7.0	21	93	61	− 91	276	29	− 88	2020/10/30 11:51
Mainshock AFAD	26.794	37.902	Mw 6.9	17	95	43	− 87	270	46	− 91	2020/10/30 11:51
			Ml 6.6								
Aftershock USGS	26.825	37.832	M 5.3	10	284	52	− 73	78	41	− 110	2020/10/30 15:14
Aftershock USGS	26.839	37.848	M 4.8	1.7	103	36	− 89	282	54	− 91	2020/10/31 05:31
University of Athens	26.723	37.875	Mw6.7	11	76	41	− 101	270	50	− 81	2020/10/30 11:51

Sources of data: USGS (https://earthquake.usgs.gov/earthquakes/eventpage/us7000c7y0/executive), AFAD (Disaster and Emergency Management of Turkey) (https://www.afad.gov.tr/izmir-seferihisar-depremi-duyuru-79-16112020---1800), University of Athens (http://www.geophysics.geol.uoa.gr/stations/gmaps3/eventpage_leaf.php?scid=nkua2020vimx&lng=en).

## Deformation of the upper plate

**Figure 1:**
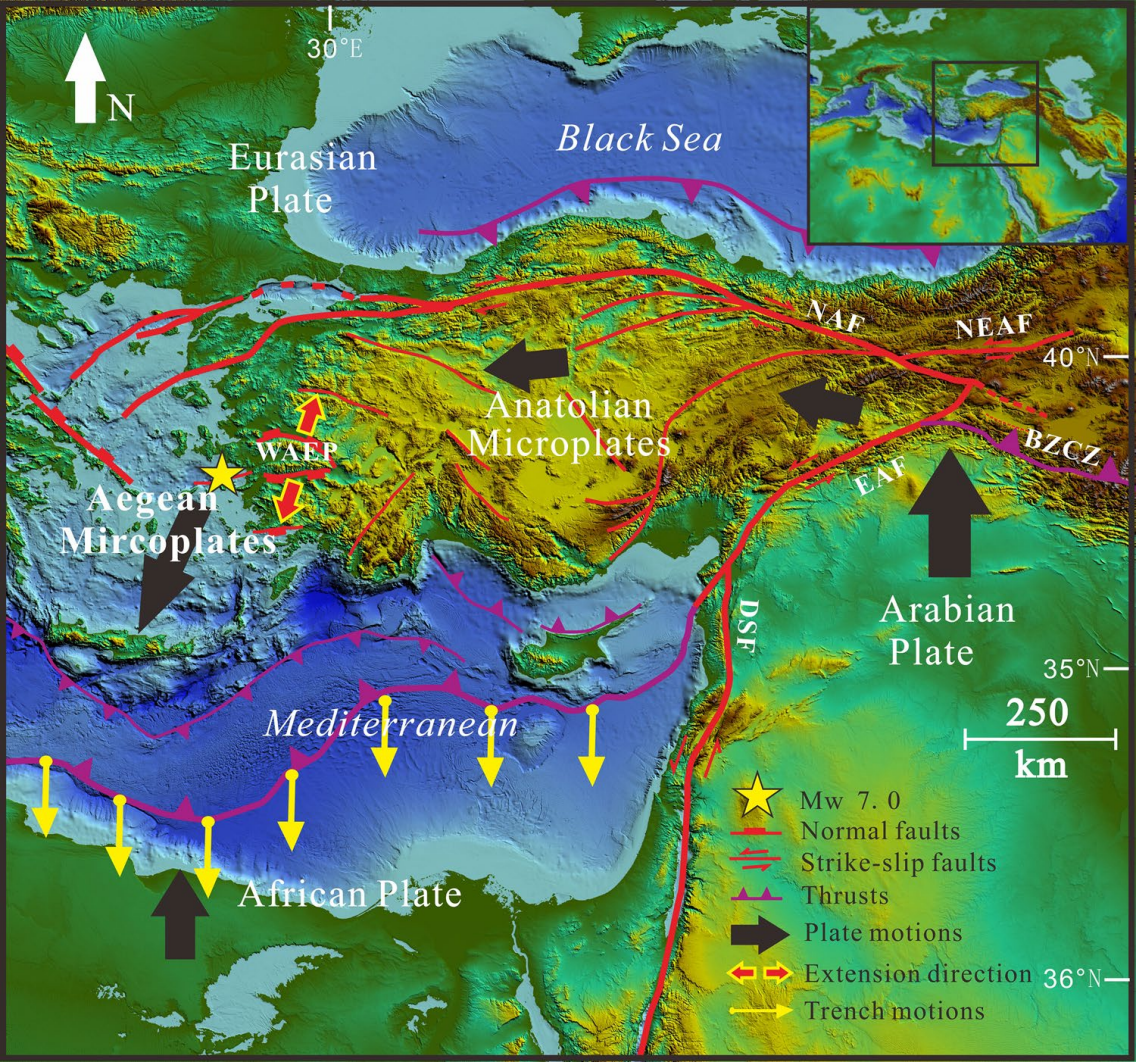
Tectonic background of Aegean and Anatolia^2,3^. *WAEP* Western Anatolian extension province; *NAF* North Anatolian fault; *EAF* East Anatolian Fault; *DSF* Dead Sea Fault; *BZCZ* Bitlis-Zagros collision zone. Dashed white line outlining rectangle represents the area of Fig. 2b. Bold black arrows indicate direction of motion of plates, yellow arrows attached to trench indicate direction of trench and slab rollback. Red arrows in western Anatolia indicate direction of extension of upper plate, and yellow star is location of the Mw 7.0 Samos earthquake of October 30, 2020.

Geologically, the area is part of the upper plate above the Hellenic subduction zone forming the Aegean volcanic arc and its continuation in Anatolia (Fig. 1), where the African plate is subducting beneath Eurasia, contributing to the ongoing final closure of the Tethyan seaways^5–10^, but in the Aegean and Anatolia of western Turkey, the tectonic forces are highly complex^11,12^. As Arabia is already colliding with Eurasia in the east (Fig. 1), some parts of Anatolia are escaping sideways to the west along the North and East Anatolian fault systems^7,8^, in a process called tectonic escape. At the same time the African plate is being subducted to the north beneath the western part of Anatolia, yet the subducting African plate is being relentlessly pulled by gravity^13–15^, so the trench and subduction system is rolling back towards Africa (Fig. 1). Coupling between the subducting and overriding plates^13^ means that as the African slab retreats, it is pulling parts of the upper plate of Anatolia and the Aegean with it to the south, causing N-S extension^11,17–21^ (Fig. 1). The forearc of the system in the Aegean includes the islands of Crete, Rhodes, and Karpathos, the modern South Aegean active volcanic arc includes the volcanoes of Santorini and Milos and the extending back-arc includes the Cyclades Islands^4,19,21^ and western Anatolia. Extension has been ongoing since the Miocene^22–31^ with the Aegean subduction zone propagating southwards by up to 1000 km^32^ due to slab rollback.

**Figure 2:**
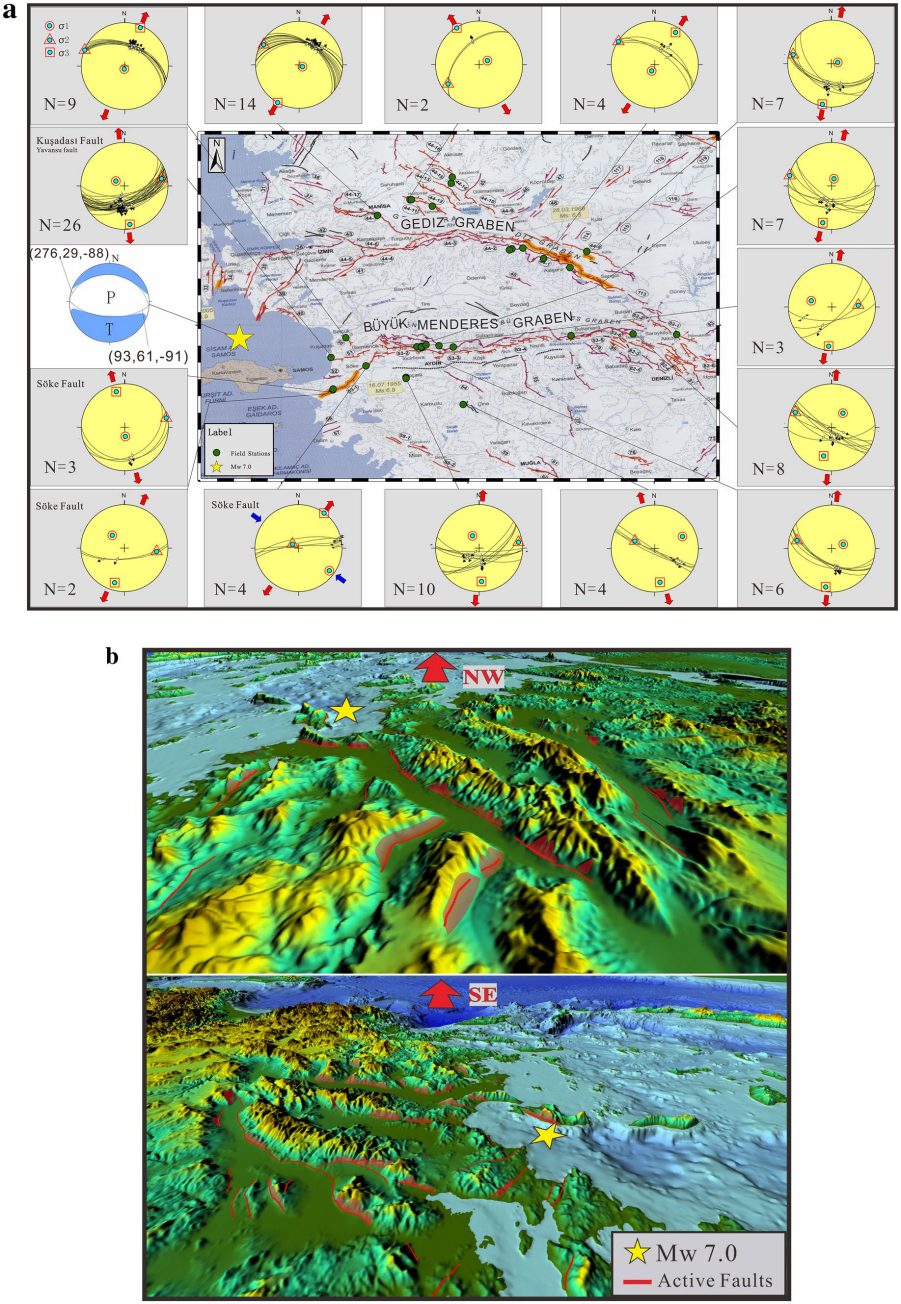
(**a**) Active fault kinematic data for the Büyük Menderes and Gediz grabens showing the orientation of our measured neotectonic faults (base map is the Active Fault Map of Turkey)^44,46^, where the lines are parallel to the geographic direction of measured fault planes. Dots show the orientation of fault-slip lineations (slickenlines) indicating the movement direction of the faults, on the fault planes. Both lines and dots are plotted on a 3-D spherical projection. These plots of fault kinematic data show the extension direction (red arrows) around the whole Menderes graben system (**b**) 3D digital elevation model (DEM) from the area outlined in Fig. 1 (dashed white box) from 2 different view directions. Note the transparent red surfaces are our interpreted triangular fault facets (Meng et al., in preparation) formed from recent faulting.

The Samos (Néon Karlovasion) Mw 7.0 earthquake occurred on a moderately N-dipping, nearly E-W striking fault plane offshore the island of Samos^33–35^, directly on-strike with the prominent Kuşadası fault system on the Anatolian coast at the westward extent of the active Büyük Menderes graben, (Fig. 2), which belongs to the Menderes graben system. Our field-based studies have focused on the kinematic, structural, exhumation, and sedimentary history of the Menderes graben system, and their associated horsts^25,26,29,36–43^, with our recent and ongoing work focused on the multi-stage slip history of the bounding faults of the Büyük Menderes Graben. The fault system includes several strands, with both synthetic south-dipping planes, and antithetic N-dipping planes, along the northern margin of the active graben system. Our structural data on active faults of these systems is summarized in Fig. 2a.

**Figure 3:**
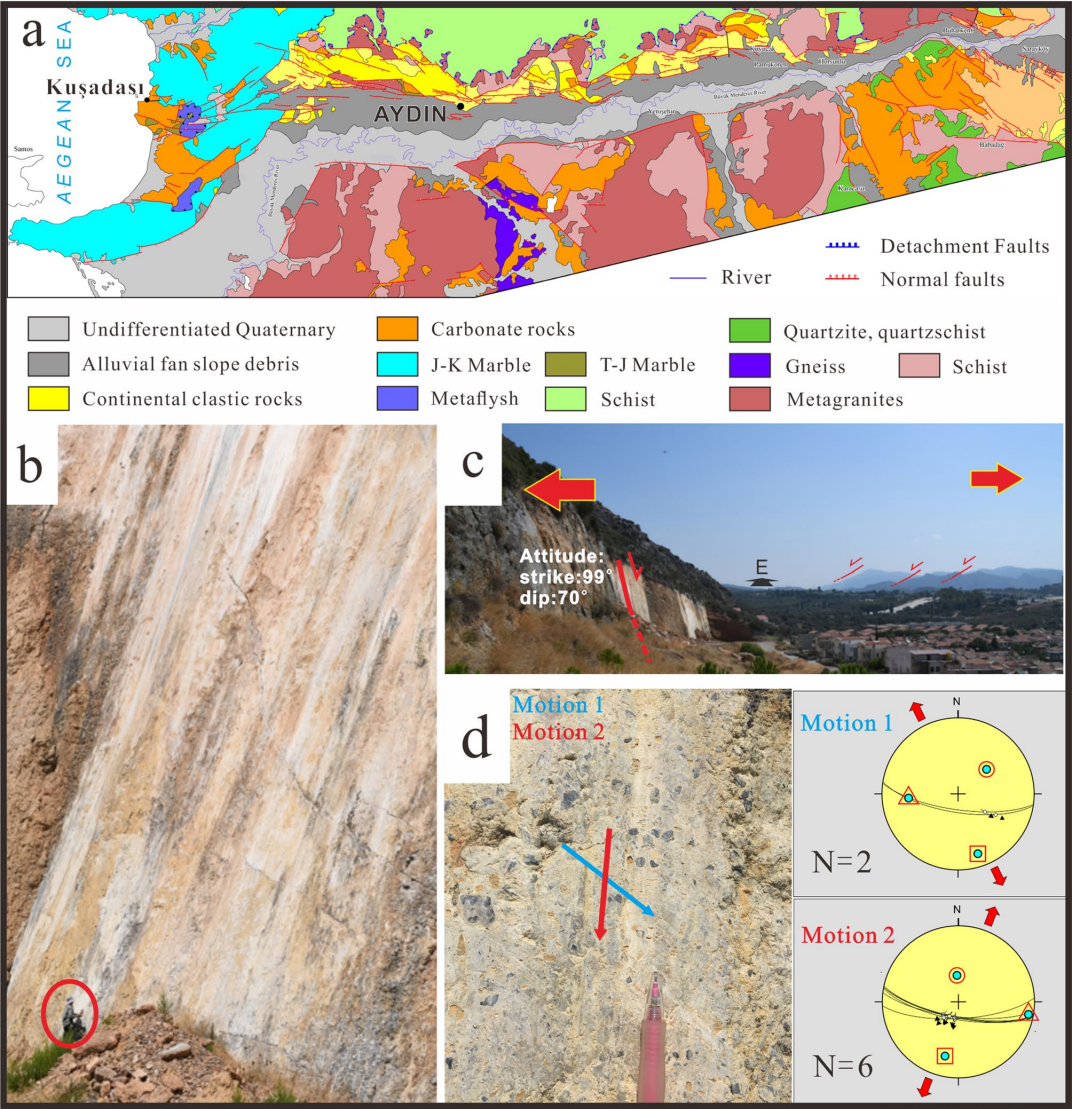
**(a)** Simplified geological map of Büyük Menderes Graben (compiled from various sources^44,45^). Photos of the Yavansu fault, Kuşadası fault system, which show (**b)** approximately 30 m highly polished slickensided surface with normal-sense slickenlines; notice the 1.8 m tall person in red circle for scale. **(c)** The synthetic and antithetic normal active faults under near N-S extension regime plunging into the graben, where human constructions are on the hanging wall exactly next to the fault surface. This presents a tremendous risk for geological hazards. Black arrow and character indicate the camera direction **(d)** fault surfaces of the Kuşadası fault showing multiple slip events and their kinematic features, Schmidt lower hemisphere equal-area projections of fault slip data, the arrows are striations^46^ (see Table 2 for details).

We report fault slip data from the Büyük Menderes and Gediz Grabens that show similar slip systems as those activated during the Mw 7.0 Samos earthquake. Along the Kuşadası fault system where the graben extends into the Aegean Sea towards Samos Island (Figs. 2b, 3b,c), both north and south dipping faults are present. The exhumed footwall rocks are composed mainly of middle to upper Miocene continental carbonate rocks and Jurassic to Cretaceous marble (Fig. 3a), which record past seismogenic slip events along the brecciated fault planes, with well-preserved slickenlines showing the fault-slip directions. These kinematic indicators generally suggest that the nearly N-S extension and can be divided into 2 groups, including a relatively older set with oblique transtensional motions, superposed by a younger, historically active set, whose slip events record nearly dip-slip motions (Figs. 2a, 3d;Table 2). Structural analyses near this fault, including on the Söke fault system and the northern margin along the Büyük Menderes Graben were conducted (Fig. 2a), where we have confirmed similar kinematic features with the main Kuşadası fault strand.

**Table 2:** Measurements of slickensides and slickenlines on high-angle normal faults of the Yavansu fault.

No	Lon	Lat	Dip direction	Dip angle	Trending	Plunge	
1	27.27	37.83	189	70	119	43	Motion1
2	27.27	37.83	188	73	124	55	
3	27.27	37.83	177	72	191	71	Motion2
4	27.27	37.83	192	73	231	69	
5	27.27	37.83	190	71	223	67	
6	27.27	37.83	191	74	195	73	
7	27.27	37.83	187	74	214	72	

The western extent of Kuşadası Fault strand and its connection with Samos Fault, on the other hand is not well established. There are seismic studies around the area, but they all include the narrow belt confined to the Turkish side of the bay, therefore they are inconclusive in terms of connecting the Kuşadası and Samos Systems. The study of Aksu et al.^47^ presents shallow seismic images. Their Fig. 10 shows there is a N dipping E-W normal fault between Samos and Kuşadası faults which is suitable to be the connection between these two. Another offshore study around the area suggests that Samos fault extends towards the Küçük Menderes Graben in the east^48^. Neither of these models present data for the exact linkage. However, based on the consistency between kinematic features of the Kuşadası fault and the focal mechanism solutions of the Samos earthquake, and our perspective on the area, we suggest that the fault responsible for October 31 Mw 7.0 earthquake is very likely to be the western continuation of the Kuşadası Fault, showing that the spectacular continental rift system of the Büyük Menderes graben extends offshore, in the more highly-extended Aegean system.. This connection needs further examination by offshore studies.

It is important to assess the past activity on active faults. By understanding the characteristics of the fault, mitigation of related geological hazards can be done to prevent damage. Mozafari et al.^43^ conducted a paleoseismology study on this fault, showing slip events on this system at 15, 8.4 and 3.6 ka, with slip magnitudes of 0.7, 0.9, and 3.1 m on the north dipping Kalafat segment, and events at 7.9, 3.4, 2.0 ka on the south dipping Yavansu strand with vertical displacements of 0.6, 3.5 and 2.6 m (M 6.5–7.1). This yields an approximate overall recurrence interval of 2400 years for slip events of ~ 1.9 m along the western on-land end of the Kuşadası fault system, but that interval is becoming shorter with time.

## Historical earthquakes and volcanic eruptions

**Figure 4:**
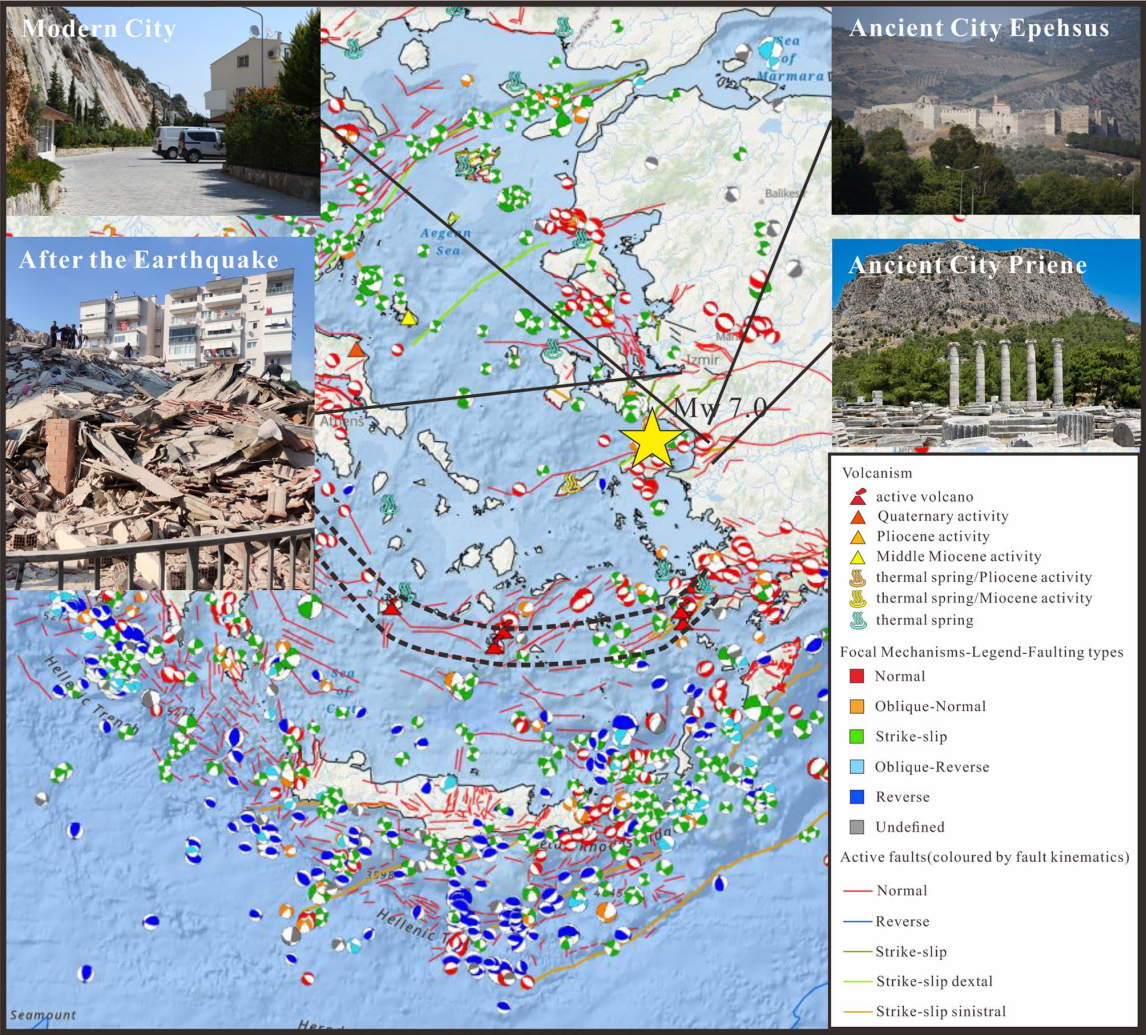
Snapshot from the online GIS platform New Seismotectonic Atlas of Greece v1.0^56,57^, presenting focal mechanisms (period 1995–June 2020, by SL-NKUA) and active faults^58^, colored by fault type, along with volcanism and hydrothermal activity. The epicenter of the October 30th 2020 main shock is presented by a yellow star. Black dash lines preset the Aegean Volcanic Arc. The inset photos shows the ancient and modern cities which suffered destructive earthquakes, note that they are all built close to active faults. The interactive GIS web application of the New Seismotectonic Atlas of Greece v1.0 is available at the following link: http://www.geophysics.geol.uoa.gr/atlas.html.

The Aegean Sea and western Anatolia region represents one of the most seismically active and most rapidly extending continental regions on the planet^3–5,19,21,26^(Fig. 4). Earthquakes, volcanic eruptions, and tsunami in the region have repeatedly caused major damage throughout history. If the relationship between plate tectonics, seismic and other geological hazard is not more greatly appreciated, history will repeat itself. Thus, we link the relatively small lesson of the Mw 7.0 Samos earthquake with some similar, yet more devastating events, some of which have changed history.

A large number of ancient cites were built close to the active fault zones, and destroyed by strong historical earthquakes^49^. For instance, the city of Priene (Fig. 4), one of the earliest Ionian settlements within the western Büyük Menderes Graben, was destroyed by a destructive earthquake in 350 BCE and rebuilt 8 km away. However, the new Priene later suffered great damages throughout history^50^. The ancient city of Ephesus (Fig. 4), an ancient Greek city on the coast of Ionia, was built in the tenth century BCE also had destroyed by earthquakes several times, while the earliest destruction can be traced to 17 CE. Relatively good historical earthquake data has been accumulated and compiled by Stucchi et al.^51^ (https://www.emidius.eu/SHEEC/), where readers are referred for further information.

Aegean earthquakes are characterized by both normal and transtensional events, with 29 earthquakes of M > 6.0 within 250 km of the Samos event in the past 100 years. These include the M 6.8 1955 Söke earthquake, M 7.7 July 1956 earthquake between Nazos and Santorini, and an associated M 7.2 aftershock. The1967 Skyros (M 6.6), 1969 Alaşehir (M 6.7), and 1970 Gediz (M 6.9) earthquakes are other significant earthquakes of the Aegean and western Anatolia extensional system^52–55^. The most recent, significant event before the Samos Earthquake was a M 6.6 earthquake on July 20, 2017 near Bodrum, on the southwest coast of Turkey (https://earthquake.usgs.gov/earthquakes/eventpage/us20009ynd/executive). These events show that faults of this area can generate earthquakes of larger magnitude than most other extensional systems around the world.

The South Aegean active volcanic arc formed along the convergent plate boundary of the northward subducting African plate underneath the active margin of the Eurasian plate^6,17^, with some historically volcanoes, such as Methana volcano at the western edge of the volcanic arc, Milos and Santorini volcanos in the central part, and Nisyros at the eastern edge near the Turkish coast, which have caused tremendous catastrophes, perhaps rising to some of the most significant in human history, together with the earthquakes^59,60^.

## Linking deep subduction, slab rollback, and upper plate extension

**Figure 5:**
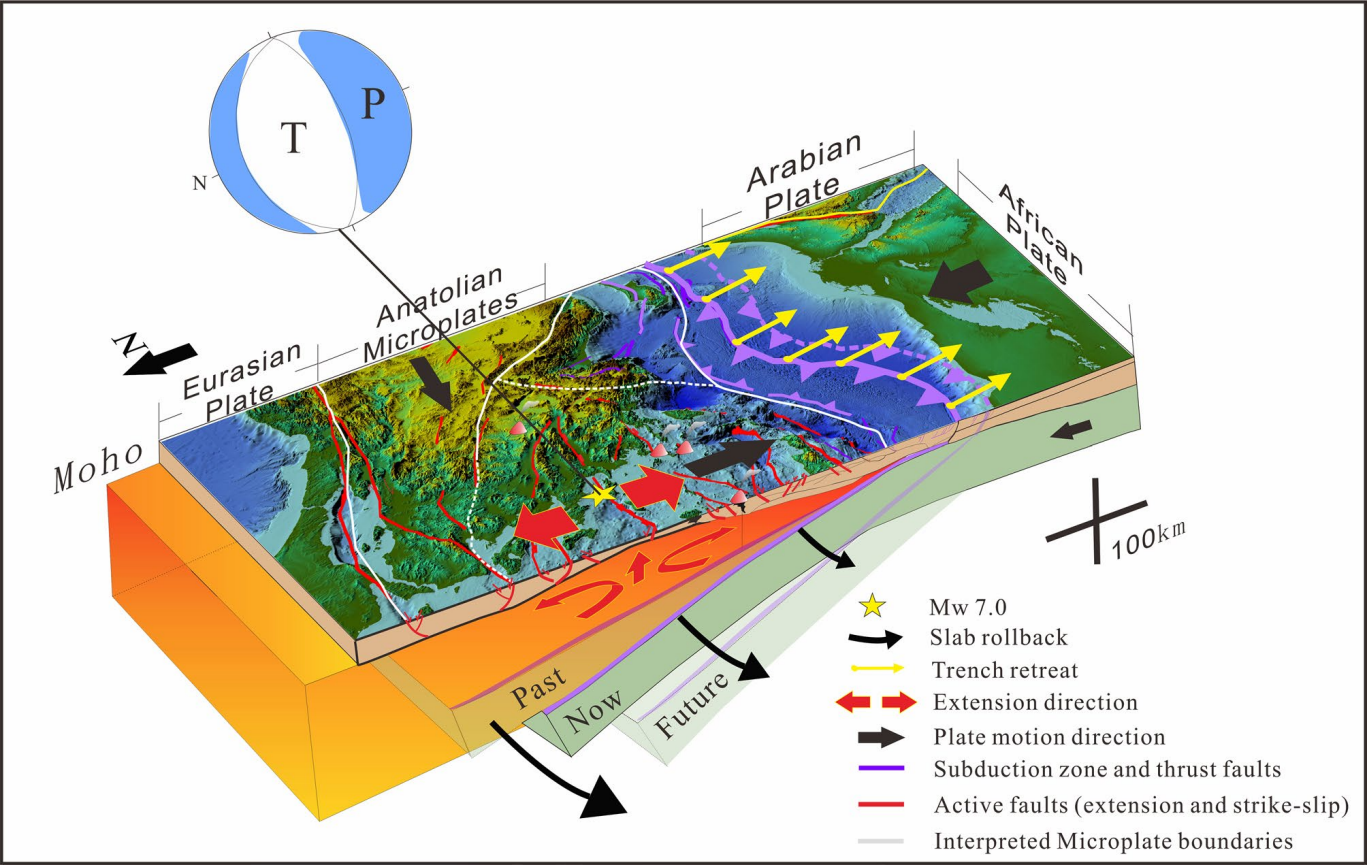
Tectonic model explaining the forces that triggered the Samos earthquake. The African plate is subducting to the north in the Hellenic and Cyprus trenches, the trench and upper plate are retreating as the slabs roll back to the south, exposed in the Cycladian Islands of the Aegean. The upper plate forearc is coupled to the rolling-back lower plate, which are retreating together, breaking the upper plate into numerous small microplates, forming the Aegean and Western Anatolian extensional provinces in the upper plate.

The African lithosphere is subducting to the north in the Hellenic and Cyprus trenches, but the trench and upper plate are retreating as the slabs roll back to the south. Coupling between the upper and lower plates^16^ causes the upper plate to extend (Fig. 5), forming the Aegean extensional province, exposed in the Cycladian Islands of the Aegean, and continuous with the Western Anatolia extensional province on the Turkish mainland (Fig. 5). We relate the October 30 earthquake to the link between the subducting and retreating African plate, to the pulling apart and extension of the upper plate (Fig. 5). We show this link through our studies of the active faults of the region, and the historical record of seismicity and volcanism, and note the significance of the link between understanding risks of tectonic processes, and hazards, with historical events including some of the most cataclysmic in recorded history^59,60^. The Samos (Néon Karlovasion) Mw 7.0 earthquake occurred at a critical location for testing the links between deep tectonic subduction, the rollback of the trench system, and how it is coupled with and linked to the fragmentation of the upper plate into numerous small microtectonic blocks. While catastrophic, the earthquake presents an opportunity and demonstrates the societal need to better understand the active surface deformation and driving mechanisms of deformation in the Aegean and western Anatolia.

The Aegean and Western Anatolia represent a unique place on Earth to elucidate geologic processes changing from subduction, to the ongoing plate collision, and the transition in between, which in this case has fragmented the upper plate into numerous smaller microplates. It shows that immediately before continental collision, the overriding plate may be broken into small platelets that move laterally with respect to each other, displacing parts of formerly continuous geological features, and that this complication may be difficult to recognize in parts of the Tethyan and other orogenic belts where collision is more advanced. The rollback process of the African plate forms the geometrically complex normal fault systems, and earlier transtensional systems, responsible for the spectacular upper crustal extension and active seismicity in this area. These relationships also explain why in collision orogens, there are often small preserved “pockets” of extensional basins, fault systems, and sedimentary and volcanic deposits. Tectonic escape is moving most of Anatolia westward along the North and East Anatolian and associated fault systems, with predominantly strike-slip tectonics in eastern Anatolia, and extensional tectonics in the west. However, the boundaries between these regimes are not yet well-defined. It is imperative that further studies are conducted in various aspects including the fault systems geometries, paleoseismic records, earthquakes, and tectonic activity evaluation, to better understand the links between tectonic processes, upper crustal deformation, and better protection of society from devastating geological hazards.

## Conclusions

- The October 30, 2020 Samos Earthquake (M 7.0) was a major event mainly related to upper plate extension caused by the rollback of the subducting African Plate.
- Earthquake solutions and analysis of active fault planes in western Anatolia demonstrate this spectacular extension, and show that the modern extension was preceded on many faults by oblique extension and strike-slip motions, perhaps reflecting a change in tectonic setting from sideways escape from the Africa-Arabia collision with Eurasia, to the pure extension related to slab rollback of the African plate, and the retreat of the Hellenic trench.
- The Kuşadası and Samos faults have similar trends, kinematic features and they are formed in the same extensional system, therefore, they are probably linked in offshore areas. Further seismic studies should be conducted to improve this connection.
- Extension of the upper plate driven by slab rollback produces relatively large magnitude earthquakes, compared to other areas of continental extension, globally.
- The region is prone to continuing earthquake and volcanic hazards from historical times to modern days.
- Further detailed studies are needed in this area to better understand and predict earthquake frequency, possible locations, and to establish better building codes to protect people's lives and property.

## Data Availability

The datasets analyzed during the current study are available from the corresponding author on reasonable request.
